# Long-Term Outcomes for Young People With Substance Use Problems in Outpatient Treatment: Gender-Specific Patterns

**DOI:** 10.3389/fpsyt.2022.888197

**Published:** 2022-05-17

**Authors:** Mikael Dahlberg, Karin Boson, Mats Anderberg, Peter Wennberg

**Affiliations:** ^1^Department of Pedagogy and Learning, Linnaeus University, Växjö, Sweden; ^2^Department of Psychology, Inland Norway University of Applied Sciences, Lillehammer, Norway; ^3^Department of Psychology, University of Gothenburg, Gothenburg, Sweden; ^4^Department of Social Work, Linnaeus University, Växjö, Sweden; ^5^Department of Public Health Sciences, Stockholm University, Stockholm, Sweden; ^6^Department of Global Public Health, Karolinska Institutet, Stockholm, Sweden

**Keywords:** young people, gender differences, longitudinal, outpatient treatment, risk factors, substance use problems

## Abstract

This study presents the results of a longitudinal research project focusing on long-term outcomes among young people after initiation of outpatient treatment for substance use problems (SUP) in Sweden. Young people are defined with the age group 13–25 years. A clinical sample of 451 young people (29% girls, median age 17 years) completed a structured interview at baseline and was followed using official records one, two, and 3 years after initiation of treatment. Gender-specific patterns at intake were described and bivariate associations and logistic regressions were calculated to analyse the links between risk factors at treatment start and indications of substance use problems 3 years later. Significantly more boys than girls displayed indications of continued SUP at 3-year follow-up. More specifically, 49% of the boys vs. 35% of the girls were identified through records as still having problems with substance use. Predictive risk factors also displayed gender-specific patterns. Primary drug use frequency and age at intake predicted indications of SUP among boys but not among girls. Placement in foster care/residential homes, depression, and early drug debut had significant predictive value regarding indications of SUP among females but not among males. Girls also displayed a greater psychosocial burden at treatment start, but a more favorable treatment outcome at follow-up. Youths with a heavy risk load at treatment start (i.e., over six risk factors) did not display a greater risk of SUP at 3-year follow-up, although our results suggest that this subgroup has indications of continued problems with mental health. Consequently, future studies should further explore gender-specific treatment pathways for young people with substance use problems. Since women and girls seem to have different risk factors, co-occurring psychiatric problems and more experiences of trauma compared to men, they might need multidimensional and more comprehensive treatment interventions that run over a longer period of time.

## Introduction

A common conclusion about risk factors for future problems with alcohol and drugs is that they are largely the same, regardless of gender (1–3). However, there is extensive support for individual risk factors having dissimilar effects on problem levels and consequences in girls and boys, respectively (4–6). Although the difference between girls' and boys' alcohol and drug use has decreased the last decades, boys still use these substances more extensively and develop problems with alcohol and drugs to a greater degree (7, 8). The size of the gender gap may also vary depending on age and substance. It can be seen as a tenacious myth in substance abuse research that women with alcohol and drug problems generally have poorer treatment outcomes than men (9); meanwhile, multiple studies have demonstrated better treatment outcomes for women than for men (5, 9). This follow-up study analyses the importance of gender for how central risk factors in young people with substance use problems (SUP) predict continued problems 3 years after initiation of outpatient treatment, with a particular focus on girls/young women. In Sweden, “young people” are defined with the age group 13–25 years.

In Sweden, specialized outpatient care for young people with SUP has increased in scope and is now available in several municipalities (10, 11). One such form of outpatient care is provided by so-called Maria clinics, where social services and healthcare collaborate. Collective knowledge of effective outpatient care measures for young people with SUP is limited compared with knowledge of equivalent treatment measures for adults (12, 13), even though these measures constitute the dominant form of treatment for young people (14). Follow-up studies have demonstrated that various outpatient treatment programmes generally contribute to reduced drug and alcohol use, but there are significant differences in results between studies regarding, for example, the share of young people who remain sober or drug-free for a given period after treatment (14). Few Swedish follow-up studies have examined young people with alcohol and drug problems (15, 16), so knowledge of how young people fare after participating in various treatment initiatives in Sweden is limited.

It has proven difficult to follow up young people with psychosocial problems, as many do not want to participate or are difficult to reach after treatment has ended (17–19). Young people who do not participate in follow-ups often have difficulties in other areas as well, for example, family problems, school problems, and criminality (20). Girls are thought to be slightly more inclined to participate in follow-up studies than are boys (21, 22). At the same time, there are strategies for achieving higher retention in longitudinal studies (23). An alternative approach for this kind of study could be to use national registers to follow young people who have participated in treatment measures in order to trace their development. The extensive selection of registers in Sweden facilitates studies that could provide new and valuable knowledge.

### Follow-Up of Young People With Alcohol and Drug Problems

Treatment for young people with SUP is generally based on a goal of abstinence, even though relapse and return to drug use are relatively common (13, 22, 24). It is also the case that a relatively large proportion of treated young people begins new treatment during the follow-up period (25). In the research area, it is now also common for other outcome measures such as reduced substance use, mental illness and crime to be applied (14).

Most studies of young people who have undergone treatment for alcohol and drug problems report results after 6 or 12 months and it is more unusual with long-term follow-ups. In order to be able to investigate which risk factors in young people predict continued substance problems in the longer term, longitudinal studies are required when they are in young adulthood (7). Here, we present a selection of longitudinal studies of young people treated for alcohol and drug problems in which the follow-up times range from 1.5 to 8 years, most of which were conducted in the USA.

In a follow-up study of 232 young people (20% girls) who underwent different forms of outpatient care for problems with alcohol and drugs, half of them showed significant improvement 1.5 years after treatment (26). In one longitudinal study, 563 young people (18% girls) were followed up 3, 6, 9, 12, and 30 months after beginning outpatient treatment for problems with alcohol and drugs (27). Of these, 48% had no or low drug use at follow-up, although 18% of them were in treatment at the time. Another study followed up 144 young people (38% girls) with alcohol problems 1 and 3 years after outpatient or inpatient treatment, in order to identify trajectories relative to several background factors (28). Half of the young people displayed reduced alcohol consumption. Another study followed up 391 young people (38% girls) 3 months and 1, 3, 5, and 7 years after beginning 12-step treatment (29). Overall, 30–40% of them reported no alcohol use and ~55% no drug use at follow-up 3, 5, and 7 years after beginning treatment. In one study, MDFT (multidimensional family therapy) was compared with CBT (cognitive-behavioral therapy) in outpatient care within the framework of juvenile court, 112 young people aged 13–18 years participated and 12% were girls (30). Both treatment methods produced comparable reductions (40%) in frequency of drug and alcohol use and in other substance-use-related problems at 2-year follow-up. Another study compared BSFT (behavioral family-based treatment) with standard outpatient treatment (31). After an average of 5 years, 261 young people (21% girls) were followed up. The results indicated no differences between the methods regarding drug and alcohol use: 12% did not use drugs or alcohol, 11% used only alcohol, 5% were back in treatment, and the rest reported drug use. In a Swedish 5-year follow-up of 147 young people (59% girls) with alcohol and drug problems who came into contact with a dependency clinic, 53% of the young people still had problems with substance use at follow-up (32).

### Factors Predicting Treatment Outcome

In connection with follow-up studies, the factors that predict positive and negative outcomes are often examined (3, 33). These factors could include those present in conjunction with initiation of treatment, as well as factors connected to the treatment being administered.

According to several studies, gender and ethnicity do not generally seem to be related to treatment outcomes (12, 14, 28, 34), although some follow-up studies find better outcomes in young women (29). Early debut of substance use is also a well-known risk factor for continued problems (35, 36). The severity of substance use at initiation of treatment is clearly linked to outcomes (14). Simultaneous mental health problems have been shown in several studies to predict worse outcomes (26, 27, 32, 34, 36); other studies, however, do not find differences in outcomes in young people with comorbidity (12, 14, 26). Parental substance abuse and neglect may be related to continued substance use in conjunction with follow-up (28, 32). Problems at school are also a significant risk factor (24). Factors that contribute to a greater degree to relapse after concluded treatment are spending time with friends who use alcohol and drugs or lack of extracurricular activities (24, 34). Criminality can also covary with substance use problems at follow-up (26, 32).

Treatment factors shown to affect relapse rate are low motivation, lack of parental involvement, and interrupted or shorter periods in treatment (12, 24, 29, 34, 37). According to one review, the first month after completed treatment is thought to entail the greatest risk of substance use relapse (34).

Overall, the results of the reported follow-up studies are consistent with research reviews concluding that 30–50% of young people relapse into drug use after outpatient treatment (13, 24). As seen from this review, knowledge about long-term outcomes after outpatient treatment is limited in terms of both follow-up studies and predictive factors. The majority of the studies are from the USA, with small and in some cases specific samples where the proportion of girls is generally low. This also applies to the studies investigating factors predicting continued SUP. This means that there are not usually analyses by gender, so the norm is boys/men.

### Aim

This article presents the results of a longitudinal/prospective study of young people with SUP in Sweden who undergo outpatient treatment, based on data taken from official registers. It aims to describe and analyse indications of continued SUP and gender-specific risk patterns in predicting continued problems 3 years after initiation of treatment.

## Methods

This study was conducted within the framework of the research project Treatment Research on Adolescents at the Maria clinics (TRAM). The central aim of TRAM is to examine young people's change trajectories regarding alcohol and drug use, mental health, and social situation, as well as how specific risk and protective factors affect outcomes for various groups after outpatient treatment. The study has been ethically approved (Ref. no. 2015/160-31). The project combines data from structured interviews with young people at intake and data from various registers at follow-up 1 year after baseline. Similar strategies have been successfully used in several Swedish studies to follow up children and young people placed in various forms of institutional care or sentenced to custodial care or imprisonment (38–40).

### Participants

Initial data were collected at Maria clinics in 12 Swedish cities, including Stockholm, Göteborg, and Malmö. These clinics are specialized outpatient units for young people with SUP and are operated in cooperation with social services and the healthcare system. The clinics offer various forms of individualized and/or manual-based treatment of alcohol and drug use disorders. The average episode of care is 4–6 months (41). The outpatient clinics are primarily aimed at young people aged 13–21. All young people aged 15 years and above who initiated contact with the Maria clinics in 2016 were invited to participate in the study; 932 individuals were informed and asked about participation in the study by the therapist in question and 469 chose to participate. Consent from parents or guardians is not required in Sweden when you are 15 years old. No register data were available for 14 individuals due to incomplete personal identification numbers or migration out of Sweden, and four youths had died during the follow-up period. Thus, a total of 451 young people participated in the 3-year follow-up study reported here. The age of the young people who make up the study sample has the age range 13–25.

### Non-response

A non-response analysis shows that the study group (451 individuals) had somewhat more serious substance use problems than did the group (477 individuals) opting not to participate in the study. The study group consisted of 29% girls, while the non-response group was 22% girls. The mean age was 18 years in both groups. Regarding primary drug, both groups reported similar patterns: in the study group, 77% used cannabis as the primary drug, 14% alcohol, and 9% other drugs; in the non-response group, the proportions were 79% cannabis, 13% alcohol, and 8% other drugs. There were significant differences in other variables related to substance use, and the study group generally had more serious SUP than did the non-response group in terms of higher drug use frequency (49 vs. 41%), greater extent of mixed substance use (38 vs. 26%), and a larger proportion with previous substance abuse treatment (31 vs. 20%). These results differ from those of earlier follow-up studies, in which, in contrast, groups that opted not to participate often had more serious drug problem (21). The differences can likely be partially explained by the somewhat larger proportion of girls—who generally have higher psychosocial loads—in the study group (40).

### Measures and Outcomes

When the treatment process began, initial data collection began via interviews based on the UngDOK interview. The purpose of this intake interview is to identify problems, needs, and current situation to enable relevant assessment, planning, and delivery of treatment. The semi-structured interview contains 75 questions in the following life domains: housing and financial support, employment, alcohol and drugs, treatment history, criminality, childhood, exposure to violence, family and relationship, and physical and mental health. Scoring of variables at baseline in the UngDOK interview was dichotomous (yes = 1/no = 0). Measures at intake have been previously described and the interview method has satisfactory reliability and validity (42).

The outcome measures used to analyse treatment results were based on experience gained in earlier studies and provided a multifaceted and reliable picture of the young peoples' progress (40). Data that indicated SUP at 3-year follow-up were taken from several different national registers. Incidence of substance use disorders (according to ICD-10) in connection with outpatient and inpatient physical, psychiatric, and addiction care was obtained from the National Board of Health and Welfare's Patient Register. Information about medication for alcohol and drug use disorders was found in the National Board of Health and Welfare's Pharmaceutical Register. The incidence of compulsory care for substance use disorders was taken from the National Board of Health and Welfare's Compulsory Care Register. Information on substance use–related criminality, such as drugs offenses or drink driving, was found in the Processed Offenses register kept by the Swedish National Council for Crime Prevention. Incidence in any of these registers were coded 1 = “Yes, indication of continued SUP”. No incidence was coded 0 = “No indication of continued SUP.”

### Statistical Analyses

Chi-square testing of independence was used to compare frequencies between girls' and boys' reports regarding variables indicating SUP at 3-year follow-up (primary outcome variable) and general risk factors at treatment start. Effect sizes were calculated using Cramér's V and can be interpreted as weak (<0.20), moderate (0.20–0.39), and relatively strong (0.40–0.59), according to Rea and Parker (2014). Bivariate associations were calculated between risk factors and indications of SUP at 3-year follow-up. Logistic regressions were used to separately describe the predictive value of the risk factors, with indication of SUP as the outcome. Nagelkerke's quasi *R*^2^ was used to determine model fit in terms of percentage of explained variance. This was done with and without controlling for gender, age, and drug use frequency (of the primary drug). In addition, gender-stratified analyses were conducted to investigate potential gender-specific risk patterns. Furthermore, logistic regression analysis was conducted to investigate the impact of cumulative risk load at treatment start on SUP at 3-year follow-up. To reduce the possibility of spurious significances arising due to multiple testing, the *p*-value of 0.05 must be interpreted with caution. SPSS 26 was used for all statistical analyses.

## Results

### Gender Differences at Intake

Table 1 presents the basic characteristics of the study group, divided by girls and boys. The average age of both girls and boys was 18 years at the time of treatment start. Most of the young people lived with their parents and were in compulsory school or upper-secondary school. Girls had severe conflicts with their parents to a greater extent. Boys had regular extracurricular activities more often. There were significant gender differences regarding drug use. Cannabis was more likely to be the primary drug for boys than for girls, while a greater share of girls said alcohol was their main drug. Significantly more girls than boys engaged in risky alcohol consumption. Boys were convicted of crimes to a significantly greater extent, while the share of girls having ongoing contact with psychiatric care was significantly greater. Significantly, fewer girls than boys displayed indications of continued SUP at 3-year follow-up. More specifically, 46 of the girls (35%) vs. 156 of the boys (49%) were identified through records as still having SUP.

**Table 1:** Descriptive variables at treatment intake and indication of substance use problems at 3-year follow-up.

	**Total**	**Girls**	**Boys**	***P***
	***n* = 451**	***n* = 132**	***n* = 319**	
**Intake**				
Age m (SD)	17.9 (2.6)	17.7 (2.7)	18.0 (2.6)	ns
Live with parents (%)	72	70	73	ns
Serious conflicts with parents (%)	36	48	31	0.001
Attends school (%)	69	68	69	ns
Participation in extracurricular activities (%)	40	31	43	0.048
Risky alcohol consumption (%)	48	57	44	0.012
**Primary drug (%)**				
Cannabis	77	65	82	0.000
Alcohol	14	22	10	0.001
Other drugs	9	13	8	ns
Usage frequency 2–3 days/week or more (%)	49	52	48	ns
Mixed substance abuse (%)	38	43	36	ns
Previous substance abuse treatment (%)	31	27	32	ns
Ever convicted of crime (%)	33	20	38	0.000
Victim of crime (%)	51	48	52	ns
Experiences serious physical health problems last 30 days (%)	15	14	15	ns
Ever treated in psychiatric care (%)	21	30	17	0.001
**Follow-up 3 year**				
Indication SUP (%)	45	35	49	0.006

*Data stated as percentages. Gender differences tested using the Chi^2^ test (ns = not significant)*.

### Predictive Factors

Furthermore, bivariate associations and predictive values of the risk factors, with and without controlling for gender, age, and primary drug use frequency, regarding the outcome variable indications of SUP at 3-year follow-up are presented in Table 2. Placement in foster care/residential home and early drug debut had significant predictive value regarding indications of continued SUP, both separately and combined with other risk factors [Model 1: $\chi_{\left[10\right]}^{2}$ = 20.971, Nagelkerke's quasi *R*^2^ = 0.061]. Early drug debut continued to display significant predictive value when the covariates gender, age, and primary drug use frequency were included [Model 2: $\chi_{\left[13\right]}^{2}$ = 41.963, Nagelkerke's quasi *R*^2^ = 0.119]. Regarding the significant predictors, gender effects were found for early drug debut, i.e., girls 29 vs. boys 20% [$\chi_{\left[1\right]}^{2}$ = 4.092, *p* = 0.043, Cramér's V = 0.095], but not for placement in foster care/residential home. Model 2 also showed that the three covariates in themselves were significant factors predicting outcomes, i.e., age: OR = 0.91 (95% CI = 0.83–1.00), *p* = 0.038; gender: OR = 0.50 (95% CI = 0.32–0.79), *p* = 0.030; and primary drug use frequency: OR = 2.10 (95% CI = 1.38–3.22), *p* = 0.001. Therefore, new analyses stratified by gender were conducted to explore potential gender-specific patterns regarding risk factors and continued SUP 2 years after initiation of treatment (see Table 3).

**Table 2:** Bivariate associations and logistic regression analyses of risk factors regarding indications of SUP 3 years after initiation of treatment.

	**Bivariate associations**	**Model 1**	**Model 2**
			**Full model**
	**OR (95% CI)**	**OR (95% CI)**	**OR (95% CI)**
1. Lack of occupation	1.02 (0.63–1.64)	0.93 (0.56–1.56)	0.93 (0.53–1.62)
2. Problems at school	1.26 (0.82–1.94)	1.29 (0.80–2.05)	1.29 (0.80–2.08)
3. Placement in foster care/residential home	1.77 (1.11–2.81)^*^	1.72 (1.05–2.80)^*^	1.63 (0.99–2.70)
4. Problems in childhood environment	1.10 (0.75–1.60)	1.13 (0.73–1.73)	1.05 (0.68–1.62)
5. Early drug debut	1.79 (1.14–2.80)^*^	1.70 (1.05–2.71)^*^	1.92 (1.17–3.14)^*^
6. Delinquent peers	1.45 (0.91–2.31)	1.42 (0.88–2.29)	1.26 (0.76–2.08)
7. Exposure to violence	0.87 (0.60–1.28)	0.83 (0.54–1.27)	0.89 (0.57–1.39)
8. Depression	0.77 (0.52–1.15)	0.70 (0.45–1.10)	0.76 (0.47–1.22)
9. Violent behavior	1.21 (0.77–1.89)	1.44 (0.88–2.35)	1.44 (0.87–2.38)
10. Traumatic events	0.81 (0.55–1.20)	0.70 (0.45–1.09)	0.79 (0.50–1.25)

* *p <0.05.*

^**^p <0.01.

*Odds ratios and confidence intervals are presented (n = 451).*

*Model 1 includes risk factors 1–10 and Model 2 includes risk factors 1–10 as well as age, gender, and primary drug use frequency at intake*.

**Table 3:** Bivariate associations between risk factors and indication of SUP 3 years after initiation of treatment.

	**Girls (*****n*** **=** **132)**			**Boys (*****n*** **=** **319)**		
	**Bivariate associations**	**Model 3a**	**Model 4a**	**Bivariate associations**	**Model 3b**	**Model 4b**
			**Full model**			**Full model**
	**OR (95% CI)**	**OR (95% CI)**	**OR (95% CI)**	**OR (95% CI)**	**OR (95% CI)**	**OR (95% CI)**
1. Lack of occupation	0.77 (0.28–2.16)	0.86 (0.28–2.62)	0.82 (0.26–2.57)	1.06 (0.61–1.84)	0.87 (0.47–1.59)	0.99 (0.51–1.91)
2. Problems at school	1.26 (0.50–3.16)	1.17 (0.41–3.32)	1.18 (0.41–3.40)	1.34 (0.82–2.21)	1.33 (0.78–2.27)	1.29 (0.75–2.23)
3. Placement in foster care/residential home	2.25 (0.96–5.26)	2.94 (1.13–7.67)^*^	2.85 (1.09–7.50)^*^	1.64 (0.94–2.86)	1.59 (0.87–2.90)	1.51 (0.81–2.79)
4. Problems in childhood environment	0.83 (0.40–1.75)	0.84 (0.35–2.01)	0.80 (0.33–1.96)	1.26 (0.81–1.97)	1.26 (0.76–2.08)	1.18 (0.70–1.98)
5. Early drug debut	2.48 (1.14–5.40)^*^	2.30 (1.01–5.24)^*^	2.37 (1.01–5.54)^*^	1.72 (0.98–3.02)	1.53 (0.84–2.79)	1.68 (0.90–3.12)
6. Delinquent peers	1.00 (0.39–2.56)	1.14 (0.41–3.22)	1.10 (0.38–3.21)	1.61 (0.93–2.77)	1.55 (0.88–2.72)	1.37 (0.76–2.46)
7. Exposure to violence	1.04 (0.47–2.30)	1.37 (0.51–3.70)	1.34 (0.49–3.65)	0.90 (0.58–1.41)	0.80 (0.49–1.30)	0.84 (0.51–1.40)
8. Depression	0.53 (0.25–1.13)	0.32 (0.12–0.85)^*^	0.32 (0.12–0.84)^*^	0.97 (0.60–1.57)	0.91 (0.53–1.56)	1.00 (0.56–1.78)
9. Violent behavior	1.25 (0.54–2.86)	2.15 (0.81–5.74)	2.20 (0.81–5.99)	1.23 (0.72–2.10)	1.36 (0.75–2.49)	1.33 (0.72–2.47)
10. Traumatic events	1.05 (0.52–2.16)	1.32 (0.56–3.09)	1.29 (0.55–3.04)	0.81 (0.50–1.32)	0.62 (0.36–1.07)	0.70 (0.40–1.24)

* *p <0.05.*

^**^p <0.01.

*Analyses stratified by gender.*

*Models 3a and 4a include risk factors 1–10 and Model 3b and 4b risk factors 1–10 as well as age and primary drug use frequency at intake*.

### Gender Differences in Predictive Factors

The gender-stratified analyses showed that placement in foster care/residential home and early drug debut, along with depression, had predictive value in the female group [Model 3a: $\chi_{\left[10\right]}^{2}$ = 15.370, Nagelkerke's quasi *R*^2^ = 0.151; Model 4a: $\chi_{\left[12\right]}^{2}$ = 15.726, Nagelkerke's quasi *R*^2^ = 0.119]. A quite different pattern emerged among males, as covariates such as age and primary drug use frequency at treatment start had distinctive predictive effects regarding continued SUP among boys, but not among girls, i.e., age: OR = 0.89 (95% CI = 0.79–0.99), *p* = 0.029, and primary drug use frequency: OR = 2.41 (95% CI = 1.47–3.94), *p* = 0.000; Model 3b [$\chi_{\left[10\right]}^{2}$ = 13.393, *p* = 0.203, Nagelkerke's quasi *R*^2^ = 0.055] and Model 4b [$\chi_{\left[12\right]}^{2}$ = 28.026, *p* = 0.005, Nagelkerke's quasi *R*^2^ = 0.112].

### Cumulative Effect

Table 4 shows the effect of cumulative risk linked to indications of substance use problems at 3-year follow-up. No significant effects were found for the uncontrolled model [Model 5: $\chi_{\left[2\right]}^{2}$ = 1.028, Nagelkerke's quasi *R*^2^ = 0.003]. However, when controlling for gender, age, and primary drug use frequency, Model 6 was significant [$\chi_{\left[5\right]}^{2}$ = 26.854, Nagelkerke's quasi *R*^2^ = 0.077]. In this model, all three covariates contributed significantly, i.e., age: OR = 0.91 (95% CI = 0.83–1.00), *p* = 0.038; gender: OR = 0.50 (95% CI = 0.32–0.79), *p* = 0.030; and primary drug use frequency: OR = 2.10 (95% CI = 1.38–3.22), *p* = 0.001. Further analyses stratified by gender were conducted to explore potential gender-specific patterns. The cumulative risk also lacks predictive value for both girls and boys at 3-year follow-up. Nevertheless, the results indicated that age and primary drug use frequency were significant predictors among boys, i.e., age: OR = 0.86 (95% CI = 0.78–0.95), *p* = 0.003; and primary drug use frequency: OR = 2.58 (95% CI = 1.59–4.18), *p* = 0.000. The same pattern was not evident among girls.

**Table 4:** Odds ratios and confidence intervals for the association between adolescent cumulative risk and indication of substance use problems 3 years after initiation of treatment (*n* = 451).

	**Model 5**	**Model 6**
		**Full model**
	**OR (95% CI)**	**OR (95% CI)**
0–2 risk factors (31%) ref	1	1
3–5 risk factors (49%)	0.76 (0.45–1.29)	0.60 (0.34–1.07)
6–10 risk factors (21%)	0.85 (0.52–1.38)	0.72 (0.43–1.20)

*Model 4 includes the level of cumulative risk as well as age, gender, and primary drug use frequency at intake.*

^*^p <0.05.

^**^p <0.01.

The group with the highest cumulative risk had a lower risk of continuing indication of SUP at 3-year follow-up. To test this, cumulative risk was cross-tabulated with indications of mental problems (i.e., outpatient, inpatient, or medical prescription) from the official records. The results showed that a significantly higher proportion of youth with more than six risk factors has indications of continued problems with mental health compared with those who do not have this indication (65 vs. 35%). In this group there is also an overrepresentation of girls.

## Discussion

The article presents the results of a prospective study of young people with SUP in Sweden who undergo outpatient treatment. The results are based on data taken from official registers. The study describes and analyses indications of continued SUP and gender-specific risk patterns in predicting continued problems 3 years after initiation of treatment.

When it comes to gender differences, girls and boys display similarities regarding their experiences of general risk factors at treatment start, but the results indicate that girls are more likely to have a psychosocial burden connected to mental health and risk behaviors, which is consistent with the findings of other studies (43–45). However, the boys' profile indicates a higher rate of criminality (32, 45, 46). Furthermore, this study shows that different types of risk behaviors in conjunction with start of treatment for alcohol and drug problems may have different implications for women and men on their way into young adulthood, and that the outcome may subsequently differ in relation to gender. First, the study shows that girls, to a considerably greater degree, lack indications of continued SUP compared with boys 3 years after treatment start—even though they are more psychosocially burdened than are boys at initiation of treatment, as other studies have also demonstrated (29, 47). A possible explanation is that girls mature earlier than boys of the same age (48). Many youths stop using drugs in young adulthood despite relatively extensive use as teenagers (8). Completion of school, transition into adult roles or opportunities for further education or other occupations, and changes in peers are associated with decreasing drug use (7). Another hypothesis is that women benefit more from the type of treatment that the relevant outpatient care clinics provide, in which creating trusting relationships and therapeutic conversations between care providers and young people are considered particularly important (49). Women may also have several other treatment contacts, for example, psychologists or GP's, after completing outpatient treatment.

When analyzing individual risk factors, several of them predict continued indications of SUP 3 years later. For girls, placement in a foster home/institution, early drug debut, and depression had predictive effects regarding a negative outcome. For boys, more general risk factors had an impact on outcome. Higher age at the start of treatment contact and a high frequency of use for the drug that caused the youth the most problems had clearly predictive effects on continued SUP among boys. Conversely, this means that early intervention at a younger age can predict a positive treatment outcome. The study also illustrates clear gender differences concerning several specific risk factors, which runs contrary to earlier assumptions that there are more similarities than differences between girls and boys regarding risk and protective factors (48, 50, 51). The fact that specific risk factors may have significance at different points in time—in other words, that some risk factors predict outcomes in the short term while others have more significance in the longer term—has previously been demonstrated in other studies (52, 53). It has also been demonstrated that risk factors common to girls and boys in their early teens do not apply to older youths (2, 48).

Another slightly surprising result is that the cumulative effect of risk that was evident at 1-year follow-up (54) and that was tested in this study no longer has the same significance. This result is also partly in opposition to the conclusions of several previous studies that the more risk factors there are, the more severe future problems with substance use will be (55–58). The reduced predictive capacity of cumulative risk may have several potential explanations, for example, that the strength of the prediction declines over time or that other risk factors not captured during enrolment in treatment are more important. It is also possible that models based on risk factors and protective factors are better suited to normal populations than to individuals who have already developed problems with alcohol and drugs, and who are the subjects of treatment for those problems. It could also be that the short-term outcome gradually decreases in what is called regression to the mean—in other words, some young people are at the beginning of a drug career when treatment begins, while others with longer-standing and more extensive SUP may make more progress over time (12, 14, 24). The fact that the model does not predict outcomes over a longer period may also be hopeful in a sense, in that young people with severe drug problems may also have a positive outcome. The risk factor model could be perceived as deterministic, but at the same time, many of its factors can be influenced.

### Limitations

The reported study is part of a research project on the outpatient treatment of young people with SUP in a naturalistic context, with follow-ups at 3 years. One limitation of register follow-up, however, is that certain central variables, such as frequencies of continued SUP, do not appear in official registers. At the same time, the non-response analysis shows that the study sample generally had more serious psychosocial problems than did the non-participant group. Another limitation is that CUS is not always detectable in registers, which may lead to underestimation. We deliberately chose to use a more conservative outcome measure (i.e., no register indication of SUP in the last 2 years) to be sure to establish an outcome measure with high specificity.

Another limitation of the study is that its results are not immediately generalisable to young people with SUP engaged in other types of treatment, such as compulsory care or inpatient treatment. However, a strength of the study is that the included young people represent several outpatient clinics in various Swedish cities, contributing to reasonably high generalisability concerning substance use among young people involved in such care. Combining information from structured interviews at baseline and several different register sources at follow-up produces reliable data and could be an innovative method for addressing the problem of non-response, which is common in traditional follow-up studies (18). For further research, studies are planned where existing risk factors are supplemented with other variables at both individual and structural level, such as psychiatric diagnoses and socio-economic background.

### Implications

A commonly occurring pattern in substance abuse treatment is that men or boys are overrepresented, despite the minor gender differences in drug use typically seen in normal populations. Previously, this was thought to be related to the male gender having more explicit problems with alcohol and drugs than the female gender. More recently, this explanation has been increasingly reconsidered and alternative interpretations have been proposed, for example, that the apparent gender difference instead concerns selection factors, such as the judicial system making significant referrals to substance abuse care (45, 59). This could mean that girls/women are only considered eligible for treatment at a later phase, and are thus not given adequate and timely support. Could it be that men have precedence in this area of healthcare as well? The gender difference may also be connected to a gendered socialization process in which women, to a greater extent than men, learn to discipline themselves and internalize their problems, which could help make their problems less visible to close relatives, schools, and other social institutions (4, 60). Women's SUP therefore merit more attention.

At the same time, it is thought that women or girls who begins treatment generally have more comprehensive and complex problems in multiple areas of life (44). The study clearly shows that outpatient treatment appears to provide positive outcomes, especially for girls regarding indications of SUP at 3-year follow-up. At the same time, it is important to analyse other outcomes, such as mental health problems.

Our analyses show that young people with a heavy risk load at treatment start (i.e., more than six co-occurring risk factors) do not display greater risk of SUP indication 3 years later. The fact that girls are overrepresented in this subgroup and take part in interventions for mental health may indicate that they receive help with an underlying problem that substance use is an expression of.

The study highlights the importance of identifying significant similarities and differences between girls and boys with alcohol and drug problems, as this knowledge can be of great importance for the design of both preventive measures and treatment elements. Since women and girls seem to have different risk factors, co-occurring psychiatric problems and more experiences of trauma compared to men, they might have different needs in treatment. These differences might not be adequately addressed in current substance use treatments (9). These can, for example, consist of multidimensional or more comprehensive treatment interventions that run over a longer period and complementary trauma treatment that has been shown to reduce both trauma symptoms and substance use (61). Since a large proportion of girls to a much greater extent than boys have experience of previous contacts with psychiatry, it should also be possible to draw attention to them and offer more relevant support at an earlier stage

Consequently, future studies should delve deeper into treatment pathways for young people with SUP. The study also illustrates the importance of conducting analyses by gender, in both descriptive and outcome studies, in order to obtain a more thorough knowledge of women's substance use problems and development pathways after treatment.

## Conclusions

The study identifies gender-specific patterns in the psychosocial characteristics at treatment start and in risk factors indicative of SUP. Girls displayed a greater psychosocial burden at treatment start, but a more favorable treatment outcome at follow-up. Gender and primary drug use frequency explained more than did the other risk factors. Cumulative high risk (i.e., more than six risk factors) did not predict long-term indications of SUP.

## Data Availability Statement

The raw data supporting the conclusions of this article will be made available by the authors, without undue reservation.

## Ethics Statement

The studies involving human participants were reviewed and approved by Regional Ethics Review Board, Linköping University (Ref. no. 2015/160-31). Written informed consent to participate in this study was provided by the participants' legal guardian/next of kin.

## Author Contributions

All authors listed have made a substantial, direct, and intellectual contribution to the work and approved it for publication.

## Funding

The research project was supported by Kamprad Family Foundation for Entrepreneurship, Research and Charity (4711 01 35), and Swedish Research Council for Health, Working Life and Welfare (2019-00549).

## Conflict of Interest

The authors declare that the research was conducted in the absence of any commercial or financial relationships that could be construed as a potential conflict of interest.

## Publisher's Note

All claims expressed in this article are solely those of the authors and do not necessarily represent those of their affiliated organizations, or those of the publisher, the editors and the reviewers. Any product that may be evaluated in this article, or claim that may be made by its manufacturer, is not guaranteed or endorsed by the publisher.
